# Short G-rich oligonucleotides as a potential therapeutic for Huntington's Disease

**DOI:** 10.1186/1471-2202-7-65

**Published:** 2006-10-02

**Authors:** Michael Skogen, Jennifer Roth, Sarah Yerkes, Hetal Parekh-Olmedo, Eric Kmiec

**Affiliations:** 1 Department of Biological Sciences, University of Delaware, Delaware Biotechnology Institute, 15 Innovation Way, Newark, DE 19711, USA

## Abstract

**Background:**

Huntington's Disease (HD) is an inherited autosomal dominant genetic disorder in which neuronal tissue degenerates. The pathogenesis of the disease appears to center on the development of protein aggregates that arise initially from the misfolding of the mutant HD protein. Mutant huntingtin (Htt) is produced by HD genes that contain an increased number of glutamine codons within the first exon and this expansion leads to the production of a protein that misfolds. Recent studies suggest that mutant Htt can nucleate protein aggregation and interfere with a multitude of normal cellular functions.

**Results:**

As such, efforts to find a therapy for HD have focused on agents that disrupt or block the mutant Htt aggregation pathway. Here, we report that short guanosine monotonic oligonucleotides capable of adopting a G-quartet structure, are effective inhibitors of aggregation. By utilizing a biochemical/immunoblotting assay as an initial screen, we identified a 20-mer, all G-oligonucleotide (HDG) as an active molecule. Subsequent testing in a cell-based assay revealed that HDG was an effective inhibitor of aggregation of a fusion protein, comprised of a mutant Htt fragment and green fluorescent protein (eGFP). Taken together, our results suggest that a monotonic G-oligonucleotide, capable of adopting a G-quartet conformation is an effective inhibitor of aggregation. This oligonucleotide can also enable cell survival in PC12 cells overexpressing a mutant Htt fragment fusion gene.

**Conclusion:**

Single-stranded DNA oligonucleotides capable of forming stable G-quartets can inhibit aggregation of the mutant Htt fragment protein. This activity maybe an important part of the pathogenecity of Huntington's Disease. Our results reveal a new class of agents that could be developed as a therapeutic approach for Huntington's Disease.

## Background

Huntington's Disease (HD) is an inherited disorder caused by expansions of CAG repeats (polyglutamine- polyQ) at the N-terminus, within exon 1, of the HD protein. The extent of polyglutamine expansion is correlated with the severity of the symptoms and their onset [1] while the pathology of the disease and neuronal cell death are thought to be associated with protein misfolding and protein aggregation. These aggregates are usually seen in the nucleus but can also be found in the cytoplasm [2]. Protein aggregates develop via a complex biochemical process with intermediates being visible during the process. PolyQ tracts within the pathogenic range induce a protein insolubility whereas Htt with nonpathogenic length maintains a measured degree of solubility [3,4].

Consistent with the aggregate toxicity hypothesis, inhibition of aggregate formation has been shown to have beneficial effects on the progression of HD in the R6/2 mouse model [5]. The implication of the polyQ aggregates in cytotoxicity validates them as targets for novel therapeutics. Despite the lack of details surrounding the molecular structure of the polyQ aggregates, high throughput screening for compounds that inhibit their formation have produced some promising results. Several compounds, including Congo Red [5] and Clioquinol [6], have been reported to inhibit the aggregation process in the R6/2 mouse model but their neurotoxicity tempers enthusiasm. Thus, identifying molecules that show efficacy with minimal toxicity should be an important consideration in the search for HD therapeutics.

Synthetic oligonucleotides (ODNs) provide a model category of reagents that meet some of these requirements. Oligonucleotides are synthetic polymers that are produced in highly purified quantities in a cost-effective way and the technology surrounding ODN synthesis has advanced dramatically in the last 10 years. Recently, Parekh-Olmedo *et al*. (2004) showed that certain classes of ODNs can inhibit aggregation. One of these groups is the G-rich oligonucleotide (GROs) class which have been used previously as aptamers to block protein function. Specifically, GROs have been shown to bind directly to STAT3 and interact with regions of the protein that enable dimerization [7] and in another instance, GROs have been shown to block the integration of the HIV into the host chromosome by interacting with the HIV integrase [8,9]. In both cases, the GRO forms a structure known as a G-quartet which arises from the association of four adjacent G-bases assembled into a cyclic conformation. These structures are stabilized by von Hoogstein hydrogen bonding [10] and by base stacking interactions. These molecules exhibit a very compact structure which allows them to interact productively with functionally important protein domains.

Much of the focus on developing therapeutics that block aggregate formation comes from a wealth of data associating HD pathogenesis with the presence of cellular inclusion bodies. But, recent evidence from *in vitro *[11-13] and *in vivo *[14-16] studies suggest that Htt inclusions may not be toxic to the cell or lead to neuronal degeneration. In fact, Hayden and colleagues have created an exciting mouse model that shows no long term effect of Htt inclusions on behavior or viability [6]. It may be true that inclusion bodies are neuroprotective and eliminating them may actually increase the potential for neurotoxicity.

Because of the known biological activity of GROs in interacting with specific protein domains, we tested this type of oligonucleotide in assays that measure the aggregation activity of mutant Htt fragment. We report that G-rich oligonucleotides, which form G-quartets, inhibit aggregation of mutant htt fragment in biochemical and cell-based assays. Overall, our results suggest that G-rich oligonucleotides could be used to examine the relationship between cellular aggregates and toxicity in various model systems.

## Results

### Biochemical analyses of GROs

**Figure 1:**
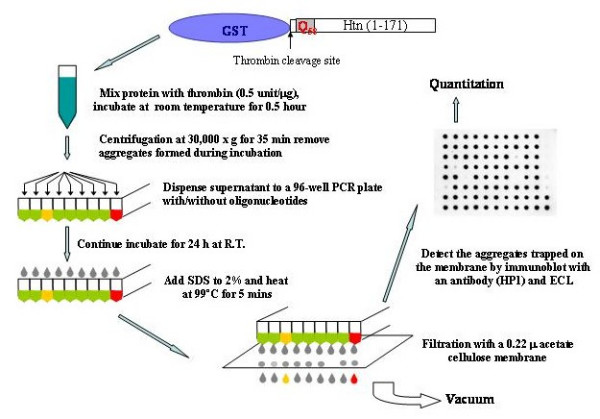
***Diagram of the biochemical model screening assay***. This diagram is taken from Wang *et al*. (2005) and illustrates the steps involved in the biochemical/immunoblotting assay used in this paper. The fusion protein GST-Q58-Htn (20 μg/ml) was mixed with thrombin (0.5 unit/μg protein) for 30 minutes and the mixture centrifuged to remove aggregated protein. The soluble protein was mixed with an oligonucleotide in a 96-well PCR plate and incubated for 24 hours at room temperature (RT). SDS was added to a final concentration of 2% and the mixture heated at 99°C for 5 minutes. Filtration through a 0.22 micron acetate cellulose membrane filter was followed by detection of aggregated Q58-Htn fragment by immunoblotting with an antibody (HP1) and ECL. Quantitation was carried out using an ImageQuant program. The blot displays both positive and negative results – positions lacking a black spot indicate that aggregation was inhibited by the oligonucleotide.

The original observation that oligonucleotides bearing random sequences reduced Htt aggregate formation [17] prompted a closer examination of a potential role for ODNs in HD therapy. We chose to utilize a biochemical/immunochemical assay system that enables rapid screening of compounds/molecules for the inhibition of aggregation [18]. In this test, oligonucleotides were mixed with purified mutant Htt fragment for 24 hours and then passed through a cellulose acetate membrane filter. The percentage of aggregates remaining on the filter was detected by immunochemistry using a primary Htt-antibody and a secondary anti-rabbit antibody conjugated to peroxidase. Signals from the SDS insoluble aggregates were scanned and quantified. A diagram of this assay, established by Wang *et al*. (2005), is presented in Figure 1. In all preparations of the mutant protein, thrombin-directed cleavage of GST-Q58Htn was allowed to proceed for 45 minutes prior to the addition of the GRO. This cleavage generates an amino terminal polyglutamine fragment consisting of 171 amino acids of the human huntingtin with tract of 58 glutamine residues [19]. The fragment is fused to GST to enable purification. We will utilize the Wang et al [19] terminology, GST-Q58-Htn to designate the protein used in this assay. The mixture was centrifuged to remove any aggregates that had already formed. Western blot analyses have shown that >95% of the GST-Q58-Htn is cleaved to completion by the thrombin [see 19]. This parameter is an important control for our study since a variety of agents are known to block the enzymatic cleavage reaction directed by thrombin [20].

**Figure 2:**
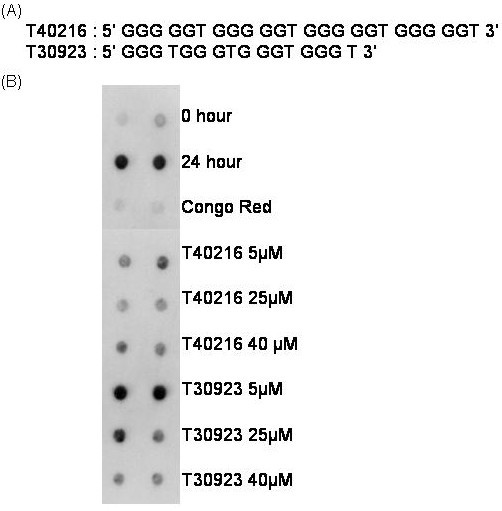
***Oligonucleotide inhibition of aggregation of mutant Htt fragment***. (A) DNA sequence of two G-rich oligonucleotides that form the G-quartet structure. (B) Dot blot analysis of T40216 and T30923 activity on aggregation. The zero (0) hour control represents reactions that were stopped immediately after addition of the protein; 24-hour reactions carried out in the absence of the oligonucleotide and stopped after 24-hours of incubation; Congo red, level of aggregation 24 hours after addition of Congo Red (10 μM).

Two known GROs were tested for inhibitory activity in the biochemical assay described above. ODN T30923 and ODN T40216 were designed by Jing *et al*. [7] and used as aptamers to inhibit the function of STAT3 protein. Both of these molecules have been determined by Circular Dichroism (CD) and NMR to have an intramolecular G-quartet structure, and similar CD spectra were seen by our lab (see below). The sequence of each is provided in Figure 2A; T30923 contains (GGGT)_4_, 16 bases in length while T40216 contains (GGGGGT)_4_, 24 bases in length.

**Figure 3:**
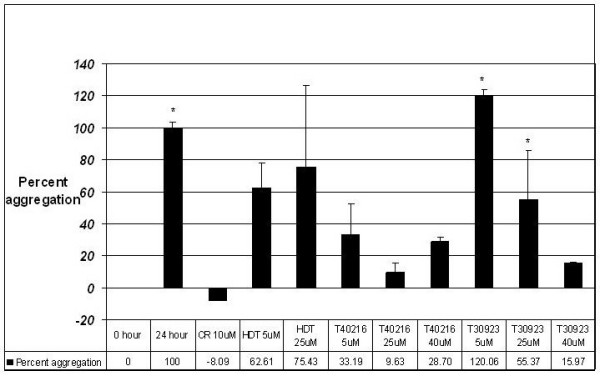
***Graphic depiction of aggregation inhibition by GROs***. Here, T40216 and T30923 and Congo Red are used: Data are presented from five independent reactions, as shown in (B) for each point with standard deviation. *, denotes significance *p *< 0.05 as compared to Congo Red (control) as determined by a one way ANOVA with Tukey's post hoc test.

These two GROs were tested in the biochemical screen for inhibitory activity of Htt fragment aggregation. GST-Q58-Htn was mixed with either T30923 or T40216 following thrombin cleavage and centrifugation (see above) and then incubated for 24 hours. Aggregation was stopped by adding SDS and 2-mercaptoethanol and heating at 99°C. The aggregates, retained on the membrane after filtration, were quantitated following immunoblotting using an image analyses program [see 19 and Material and Methods]. Three control reactions, designated 0-hour, 24-hour and Congo Red (Figure 2B), were repeated for each experiment. The 0-hour control displays the amount of aggregation at the start of the reaction, usually none. The 24-hour control reflects the amount of GST-Q58-Htn aggregation when no inhibitor is added to the mixture. The third control displays the degree of aggregation formed in the presence of Congo Red, a known inhibitor [21] functioning as the positive control in the series. As shown in Figure 2B, both T40216 and T30923 are capable of inhibiting GST-Q58-Htn aggregation with a dose response visible in the samples with T30923. Figure 3 represents the results of five experiments conducted in duplicate, followed by quantitation using ImageQuant analytical software. A statistically significant difference is observed between each GRO and the 24-hour control in each experiment.

**Figure 4:**
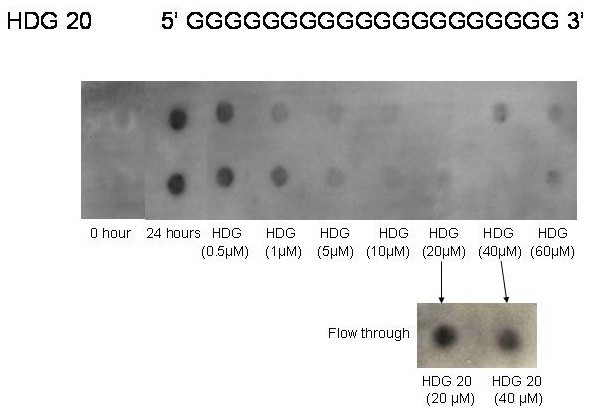
***HDG 20 inhibits aggregation of mutant Htt fragment***. HDG 20, a 20-base monotonic G oligonucleotides was tested in the assay outlined in Figure 1 (see legend) at the indicated concentration. The reaction was carried out 4 times in duplicate for 24 hours and a representative blot from the four independent experiments is shown. 0 hour, reaction mixture stopped at time zero; 24 hours, control reaction lacking oligonucleotides. (Inset), The Flow-through from filter binding reaction containing HDG 20 (20 μM or 40 μM) was placed on blotting paper, dried and processed as described in the legend to Figure 1.

**Figure 5:**
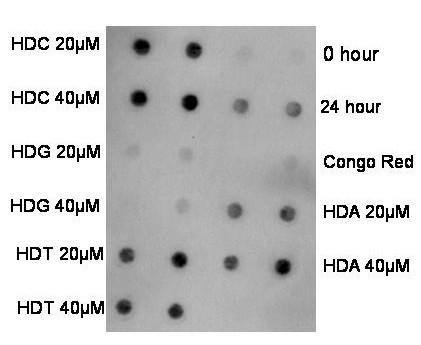
***Specificity of monotonic oligonucleotides in the inhibition of mutant Htt fragment aggregation***. Various monotonic 20-mers were tested for inhibitory activity in the assay outlined in Figure 1. HDC, 20-mer with all Cs; HDA, 20-mer with all As; HDT, 20-mer with all T's; HDG, 20-mer with all Gs. Four independent experiments were carried out in duplicate and this blot is most representative of all of the results. 0 hour, reaction stopped at zero time point; 24 hour, reaction lacking oligonucleotides, stopped at 24 hours; Congo Red, incubation with 10 μM of Congo Red for 24 hours.

**Figure 6:**
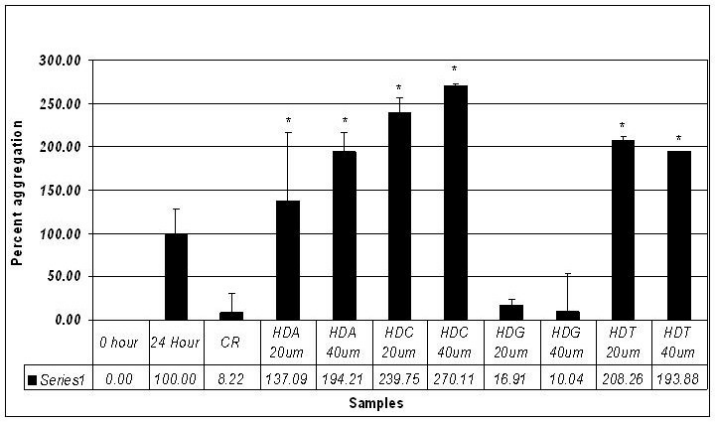
***Graphic depiction of blots carried out to test monotonic 20-mers***. Average aggregation levels, representative of 5 independent experiments with standard deviation and average values (Series 1) presented. *, denotes significance *p *< 0.05 as compared to Congo Red (control) as determined by a one way ANOVA with Tukey's post hoc test.

The effect of GROs on aggregation prompted an examination of the activity of a monotonic guanosine oligonucleotide (HDG) because this molecule can also form a G-quartet. We chose 20 bases as a compromised length of T30923 (16 bases) and T40216 (24 bases) to establish the HDG series. When a dose range of HDG was tested in the biochemical assay, inhibition of aggregation Q58-Htn fragment was readily observed (Figure 4). A significant decrease was seen at 1 μM, a much lower final concentration than the inhibitory level found with either T30923 or T40216. HDG was found to be unique in its inhibitory activity compared to other monotonic oligonucleotides. Huang *et al*. [18] demonstrated that the flow-through fraction of reactions containing inhibitors of aggregation is comprised predominantly of monomeric Htt fragments. To verify that the flow-through in reactions bearing HDG 20 contains mutant Htt fragments, we captured this fraction and placed it on blotting paper. Stacked membranes to capture monomers using the same antibody used to detect aggregates. As seen in the inset for Figure 4, a positive reaction was observed indicating the presence of mutant Htt fragment. When 20-mers of A (HDA), T (HDT) or C (HDC) were tested at 20 μM and 40 μM, no inhibition of aggregation was observed (Figure 5). Quantitation after scanning revealed a large, statistically significant difference in the activity of HDG compared to any of the other monotonic ODNs (Figure 6). Taken together, our results suggest that HDG, a 20-mer containing all G residues, is a powerful inhibitor of aggregation of Q58-Htn fragment based on the results of the immunoblotting assay.

**Figure 7:**
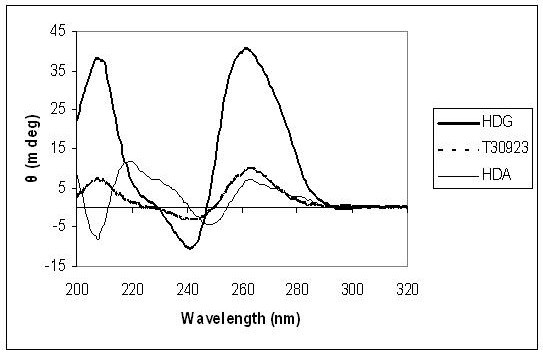
***CD spectroscopy of HDA and HDG***. CD spectra of 15 μM HDG (heavy solid line), 15 μM T30923 (light solid line) and 15 μM HDA (dotted line) in 10 mM KCl at 24°C.

CD measures differences in the absorbance of right-handed and left-handed circularly polarized light and can be used to investigate DNA helicity. G-quadruplexes can exist as antiparallel monomers, dimers or tetramers or as parallel tetramers. Traditionally, antiparallel conformations are characterized by a positive ellipticity maximum at 295 nm and a negative minimum at 265 nm. In contrast, the parallel conformation is characterized by a positive maximum at 264 nm and a negative minimum at 240 nm; however, recent results have shown some antiparallel structures to have some positive maximums at 264 nm and negative minimums at 240 nm [22-24]. HDG was characterized by CD in order to gain a perspective view of its structure. HDG was analyzed along with HDA and T30923 at 15 μM in 10 mM KCl and at 24°C. CD spectropolarity was determined using an AVIV Model 202 spectrometer with an effective range of analysis from 200 nm to 320 nm (Figure 7). HDA has an unusual maximum absorbance at 220 nm with a smaller positive absorbance at 260 nm. T30923 and HDG, however, exhibit maximum positive absorbances at 264 nm and negative minimums at 241 nm, a distinct profile that matches closely with molecules known to adopt G-quartet structures. HDG is a more effective inhibitor of GST-Q58-Htn aggregation than T30923 which is known to adopt a dimer basket G-quartet conformation [see 7] suggesting that HDG's structure is a more active conformation in our assays. Further structural studies are ongoing.

### Inhibition of aggregate formation in HEK293 cells

**Figure 8:**
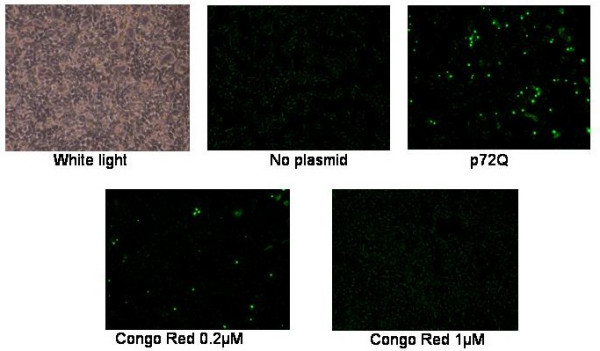
***Inhibition of aggregation in HEK 293 cells transfected with plasmid, pcDNA3.1-72Httexon1-eGFP (p72Q)***. Series of control reactions including HEK293 photographed under white light or in dark field, aggregate formation produced by p72Q and inhibition of aggregation by Congo red (0.2 μM or 1 μM) added concurrently with p72Q.

**Figure 9:**
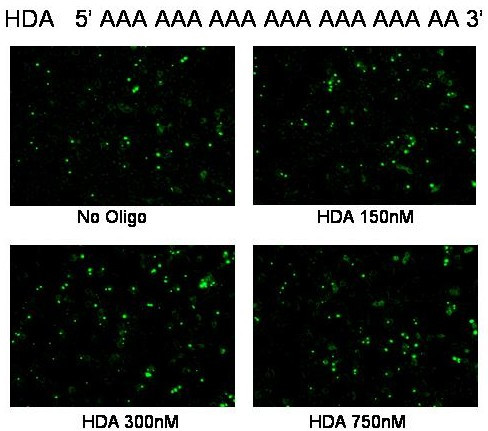
HDA was co-transfected at the indicated concentrations with p72Q and the cells were photographed 48 hours later in dark field.

**Figure 10:**
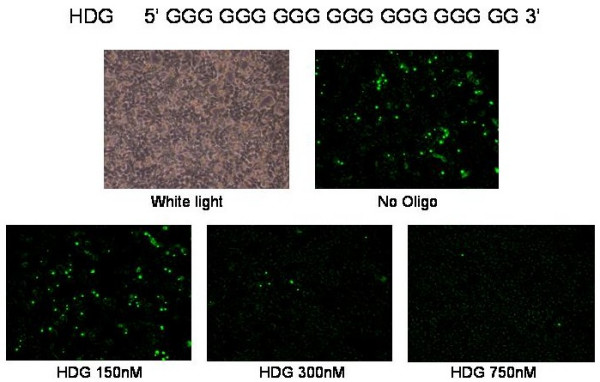
HDG was co-transfected at the indicated concentrations with p72Q and the cells were photographed 48 hours later in dark field.

**Figure 11:**
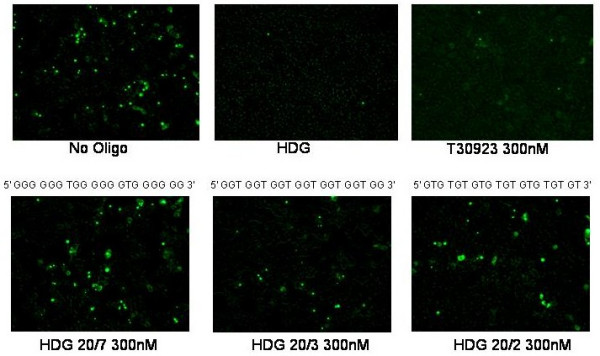
The indicated ODN was co-transfected with p72Q and the cells photographed 48 hours later under dark field. The upper left panel represents a reaction lacking oligonucleotide.

**Figure 12:**
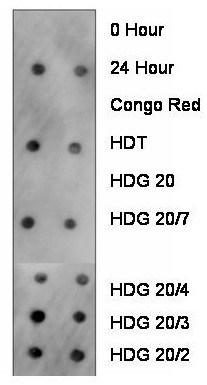
Various 20-mers with thymine modifications were tested for inhibitory activity in the assay outlined in Figure 1. HDT, 20-mer with all T's; HDG, 20-mer with all G's; HDG 20/7, 20-mer with every 7^th ^nucleotide replaced with T; HDG 20/4, 20-mer with every 4^th ^nucleotidereplaced with T HDG 20/3, 20-mer with every 3^rd ^nucleotide replaced with T; HDG 20/2, 20-mer with every other nucleotide replaced with T. 0 hour, reaction stopped at zero time point; 24 hour, reaction lacking oligonucleotide and reaction stopped at 24 hours; Congo Red, incubation with Congo Red (10 μM) for 24 hours.

Since HDG exhibited strong inhibitory activity of GST-Q58-Htn in a biochemical assay, we tested this molecule in a cell-based assay. The human embryonic kidney cell line HEK293 was transfected with plasmid pcDNA3.1-72Httexon1-eGFP (p72Q), a construct that contains a fusion gene uniting the first exon of the HD gene containing a polyQ repeat of 72 codons and the eGFP gene (J. Pearson, unpublished data). This fragment of huntingtin differs from GST-Q58-Htn in both length of polyglutamine stretch and that it is fused to eGFP rather than GST. When transfected into HEK293 cells, the gene is expressed and aggregates appear within 12 hours, reflected by the appearance of discrete green foci. Cells were photographed first under white light to verify that equal numbers of cells were present for each treatment and a representative sample is shown. eGFP foci were then imaged in the presence or absence of plasmid p72Q. In Figure 8, green fluorescent foci are evident when p72Q is present but a significant reduction is seen in cells that have also received Congo Red. Importantly, inhibition of aggregate formation is only partially inhibited when a lower dosage of Congo Red is present, demonstrating a dose-dependent effect. In Figure 9, a cell population in which HDA was co-transfected with p72Q is presented; HDA appears to have had little effect on the number of aggregates formed in these cells. As is the case in the biochemical assay (Figure 5), HDA does not appear to inhibit aggregate formation in HEK293 cells. Figure 10 illustrates the effect of HDG on aggregate formation. The white light photograph again reveals that HDG has no detectable toxic effect on cells or cell growth at 750 nM (top left panel), but a clear dose effect is seen on the number of aggregates when the level of HDG is increased (bottom panels). These observations confirm results obtained in the biochemical assay using HDG as the inhibitor. Finally, in Figure 11, a panel of photographs reveals once again that HDG is an effective inhibitor of aggregate formation but that this positive activity can be reduced significantly when T residues are inserted at the 7^th ^and 14^th ^position of the HDG 20-mer (HDG 20/7), every third base of the HDG 20-mer (HDG 20/3) or every other base of the HDG 20-mer (HDG 20/2). To validate these results and to further explore the relationship between the cell-based, and biochemical assays, we assayed HDG 20/7, HDG 20/4 and HDG 20/3 individually for activity in the immunoblot assay (see Figure 2B). These results confirm the low level of activity observed for HDG 20/7, HDG 20/3 and HDG 20/2 respectively in the cell-based assay (Figure 12). The correlation between the results obtained in the cell-based and immunoblot assay reveal a similar mode of action for the oligonucleotides. We have preliminary evidence that the reduction in aggregates observed in the cell-based assay can be confirmed when aggregates isolated from transfected cells are passed through the immunoblot assay (M. Skogen *et al*. in preparation).

**Figure 13:**
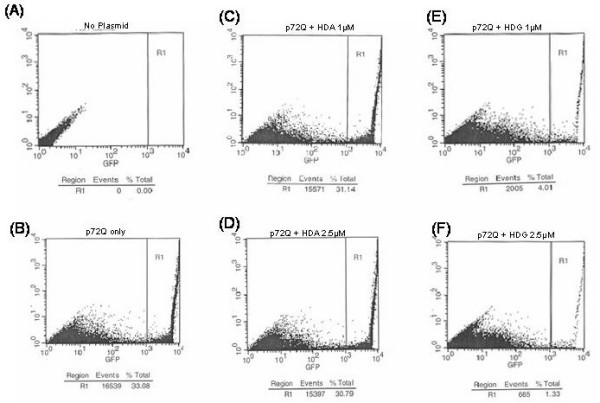
***Inhibition of mutant Htt fragment aggregation by oligonucleotides as measured by FACS***. HEK293 cells were transfected with pcDNA3.1-72Httexon1-eGFP (p72Q) and the specific ODN (HDA, HDG) at the indicated concentrations and the degree of aggregation measured by FACS after 48 hours. (A) No plasmid (p72Q); (B) only p72Q, no ODN; (C) p72Q and 1 μM HDA; (D) p72Q and 2.5 μM HDA; (E) p72Q and 1 μM HDG; (F) p72Q and 2.5 μMHDG. All ODNs are at final concentrations in the cell culture reaction. The magnitude of green fluorescence is measured on the X axis while the number of cells exhibiting that degree of fluorescence is depicted on the Y axis.

Aggregate reduction in response to the addition of HDG can also be seen using FACS analysis as the readout. Again, HEK293 cells were transfected with p72Q with or without HDG (or HDA) and the reactions were allowed to proceed for 48 hours. The cells were then processed for FACS and measured for green fluorescence. The Y axis reflects the degree or intensity of fluorescence. As seen in Figure 13A, the background is gated at the far left of the graphic whereas expression of p72Q produces a sharp peak of green fluorescence near the right edge of the profile (Figure 13B). This peak represents aggregated Htt-eGFP, scored by FACS as cells containing high intensity eGFP (aggregates). In Figures 13C and 13D, the profile of cells treated with HDA is represented and little detectable change is observed in the peak at the far right edge. Even as the level of HDA is increased from 1 μM to 2.5 μM, no significant reduction in aggregates is observed. In contrast, cells treated with HDG exhibit a very different profile (Figures 13E and 13F) as the peak representing aggregates is diminished in a dose-dependent fashion. Thus, taken together, the data suggest that HDG can inhibit aggregation formation in HEK293 cells expressing the Htt-eGFP fusion protein from plasmid p72Q.

**Figure 14:**
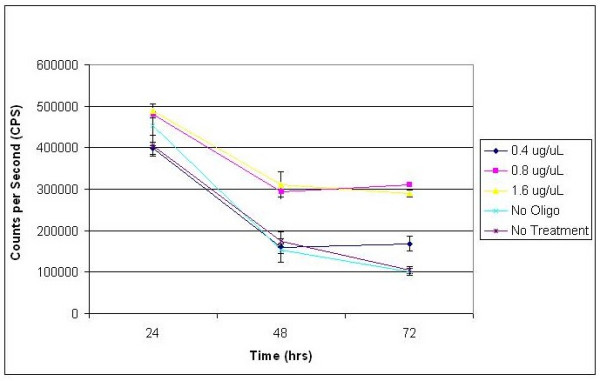
***Viability assay of PC12 cells transfected with various HDG concentrations***. The PC12 cell line, Htt14A2.6, was transfected with varying amounts of HDG in Lipofectamine 2000. The Promega CellTiter-Glo Luminescent cell viability assay was used to analyze the viability of each treatment as a function of time. One control is a mock-transfected cell culture containing lipofectamine 2000 but no oligonucleotide; whereas the no treatment control presented here indicates cells that have received neitheroligonucleotide nor lipofectamine. Statistics were performed by using the standard deviation on 27 luminescent readings for every sample at each time point.

Finally, a derivative PC12 cell line, Htt14A2.6 [25], was used to measure the capacity of HDG to improve cellular viability. This neuronal cell line is used as a standard in the field for studying the survival phenotype associated with aggregate formation. In this assay, a truncated form of Htt exon 1 (103Q) fused to enhanced green fluorescent protein (eGFP) is induced to express by addition of muristerone to the culture. After induction, cell viability decreases rapidly between 48 hours and 72 hours, respectively, as measured by a CellTiter-Glo Luminescent cell viability assay, as shown in Figure 14. The addition of increasing doses of HDG (0.4 – 1.6 μg/μl) appears to arrest the drop in viability providing some level of neuroprotection. The differences in these are statistically significant and a larger, survival study is underway to confirm and/or expand upon these results.

## Discussions and conclusion

Intracellular aggregates of Htt have long been considered phenotypic evidence of the neurodegenerative disorder Huntington's Disease. It is, however, not clear how the appearance of such inclusion bodies relates to the pathogenesis of the disease. A number of model systems have been designed to screen for therapeutic agents that can inhibit aggregation. Some of these assays measure the inhibition of fusion protein aggregation, proteins containing a fragment of Htt (here, GST-Q58-Htn) and a marker/reporter protein, often eGFP. The Htt component of this fusion protein harbors an expanded polyQ stretch.

We have examined the capacity of G-rich oligonucleotides that can adopt a G-quartet conformation to block aggregation. A well-established biochemical assay was used to examine GROs blockage of aggregation. Molecules T40216 and T30923 that are known to form intermolecular G-quartets were found to be effective inhibitors of aggregation. Both of these GROs, which were used previously to inhibit Stat3, adopt conventional G-quartet structure with the G residues (quartets) in the center and a loop domain at the top and bottom [7]. The GRO HDG, which exhibits the highest level of activity in the aggregation assays, can also adopt a stable G-quartet structure and further studies to elucidate the details of the G-quartet structure adopted by HDG are currently being performed. This molecule also may prevent or delay neurotoxicity in PC12 cells.

The GRO, HDG, is unique among monotonic oligonucleotides containing 20 bases. None of the related 20-mers, HDA, HDC or HDT show reproducible inhibitory activity in the biochemical or cell-based assays. Furthermore, HDG displays a dose response with concentrations as low as 1 μM exhibiting substantial levels of aggregate reduction. It is effective when added at the start of the Q58-Htn aggregation reaction but much less so when added after the process has begun (Skogen *et al*., in preparation). Thus, it is likely to block the nucleation phase of aggregation rather than the elongation phase, although our experiments were not designed to discriminate between these two phases [26,27].

HDG is also quite active in blocking aggregation of the Httexon1-eGFP fusion protein aggregation in HEK293 cells. In this system, the fusion protein is produced from an expression plasmid and co-transfection with HDG prevents the appearance of green fluorescent foci in a dose-dependent inhibition. Importantly, the well-known aggregation inhibitor, Congo Red, was used as a positive control and displayed effects similar to HDG. MTT viability assays reveal no cell toxicity or negative effects on cell growth as a function of oligonucleotide addition (data not shown). This result is not surprising since ODNs used as antisense or antigene therapy have been found to be practically inert in human cells with regard to cytotoxicity. A number of clinical trials using ODNs have taken place and while the efficacy of such treatments may be questioned, significant adverse effects on cells or patients were not observed. The lack of serious side effects from oligonucleotides is a virtue in the development of these molecules for use in HD patients. For example, while the effective levels for GRO actvitiy presented in this work are higher than those used for traditional pharmaceuticals, oligonucleotides are particularly well-tolerated in humans. The level herein is not unusual and levels exceeding 50 mg/kg have been found to be both efficacious and nontoxic in various antisense therapies. The higher amounts may be required because delivery to target cells or penetration into the cells may be less efficient than other drug treatments.

The potency of the G-quartet structure of HDG is demonstrated further in the activity of the modified HDGs. Using a type of reverse genetics strategy, we created several "mutant" HDGs; HDG 20/7 wherein each 7^th ^G was replaced with a T, HDG 20/3 wherein each 3^rd ^G was replaced with a T and HDG 20/2 wherein every other G was substituted with a T residue. None of these molecules were found to be effective inhibitors of aggregation. The results presented in Figure 11 most clearly illustrate the importance of the HDG G-quartet structure while support for this notion is also gained when T30923 could not fully substitute for HDG in the HEK293 assay.

The mechanism by which HDG exhibits its effect in these assays is not clear, but several paths can be suggested. G-quartets created either by natural processes or by exogenous addition, are reactive structures in the cell. They were first recognized in studies aimed at understanding the mechanism of cellular aging [28] occurring naturally near the ends of chromosomes. These telomeric structures are composed of repetitive blocks of the double-stranded DNA sequence (TTAGGG)_n _with the guanines forming an overhang at the end of the telomere. In humans, these sequence blocks can become extremely long, increasing the probability that they adopt secondary (G-quartet) structures. Such structures are known to inhibit telomerase activity and may explain why telomerase replication/activity in transformed cells is often absent or reduced [29,30].

G-quartets formed within GROs have also been shown to inhibit protein dimerization of such molecules as STAT3 [7]. They exert their activity by binding to specific domains within STAT3 with a high degree of precision. Since mutant Htt aggregation relies on a nucleation phase in which the mutant protein begin to assemble, HDG could block the transition between nucleation and elongation as aggregation (dimerization) begins. Alternatively, HDG could block other enzymes involved in the development of the pathogenic phenotype, such as caspases which cleave the native protein perhaps producing a toxic fragment [31,32]. Bates and colleagues have shown that certain GROs can bind to nucleolin in a variety of cancer cells with a high degree of specificity [33]. In all of these cases, direct interactions with cellular proteins would be required and such a reaction is a well-documented characteristic of GROs. Studies are now underway to determine which proteins are binding to HDG and if there is a difference between the composition of protein-GRO complexes in mutant and wild-type cells.

## Methods

### Biochemical aggregation assay

To analyze the inhibition of aggregation by GROs, a biochemical assay was employed (Figure 1). The fusion protein GST-Q58-Htn [19] was incubated for 45 minutes at room temperature with thrombin (1 U/1 μg protein) at a concentration of 10 μg/ml in a buffer of 50 mM Tris-HCl, pH 8, 100 mM NaCl, 2.5 mM CaCl_2_, and 1 mM EDTA, to cleave between the huntingtin and GST. As indicated by Wang *et al*. [19], this fragment consists of the amino terminal 171 amino acids with a tract of 58 glutamine residues fused to GST. The protein mix was then centrifuged at 30,000 × g at 4°C for 35 minutes to remove any aggregates that had already formed. The protein was added to wells containing 0.5–60 μM GROs or control ODNs, 10 μM Congo Red, or no treatment in the buffer detailed above with 100 mM KCl replacing NaCl. The 0-hour control was stopped immediately and after 24 hours incubation at room temperature the remaining reactions were stopped by adding 10% SDS/50 mM 2-mercaptoethanol and heating to 99°C for five minutes. The mixture was diluted in 1X PBS and then filtered through a cellulose acetate membrane (Osmonics) using the Easy-Titer ELIFA system (Pierce) followed by a 2% SDS wash. After blocking in 5% milk/1X PBS-0.05% Tween, the membrane was incubated with a specific anti-huntingtin antibody (HP1, 1:1000 dilution), followed by incubation with a peroxidase-conjugated goat anti-rabbit antibody (Sigma, 1:40,000 dilution) and chemiluminescence reagent (ECL-Plus, Amersham). Signals from the aggregates retained on the filter were scanned and quantified using ImageQuant image analysis software (Molecular Dynamics). Aggregates were quantified by optical density and statistical differences were determined by one way ANOVA with Tukey's post hoc analysis using Statistical Package for the Social Sciences (SPSS). Significance was determined by a *p *< 0.05 as compared to Congo Red (control).

### HEK-293T cell-based aggregation assay

Human embryonic kidney cells, HEK293T, were grown in low glucose DMEM supplemented with 10% FBS. Cells were seeded at 0.5 – 1 × 10^6 ^cells/well on 6-well plates. The cells were transfected with 1 μg of the plasmid pcDNA3.1-72Httexon1-eGFP (p72Q) and 150 – 750 nM GRO or control ODN using 2.5 μl Lipofectamine 2000 (Invitrogen). Forty-eight hours after transfection cells were viewed to determine the approximate number of green fluorescent foci using an Olympus IX50 microscope.

### Circular dichroism spectroscopy

Circular dichroism spectra of 15 μM oligonucleotide samples in 10 mM KCl were recorded on an Aviv model 202 spectrometer. Measurements were performed at 24°C using a 0.1 cm path-length quartz cuvette (Hellma). The CD spectra were obtained by taking the average of two scans made at 1 nm intervals from 200 to 320 nm and subtracting the baseline value corresponding to that of buffer alone. Spectral data are expressed in units of millidegree.

### PC12 viability assay

Rat pheochromocytoma cells, PC12, were grown in high glucose DMEM with 10% horse serum and 5% FBS while under selection with G418 (0.05 mg/mL) and Zeocin (0.1 mg/mL) (Invitrogen). This cell line, Htt14A2.6, expresses a truncated form of expanded repeat Htt exon 1 protein containing 1–17 amino acids and 103 polyglutamine tract fused to eGFP. The promoter was induced with muristerone resulting in the expression of the Htt exon 1 with expanded 103 CAG polyglutamine (103Q) region. Cells were seeded at 3 × 10^4 ^cells/well on a 24-well plate and transfected with a ratio of 0.8 μg HDG 20 to 2 μL Lipofectamine 2000 (Invitrogen) depending on the desired HDG concentration. After a 4-hour treatment, the transfection media was removed, whole media was added for 1-hour, and then the cells were induced using 5 μM muristerone for 24 hours. The Promega CellTiter-Glo Luminescent cell viability assay was then used. The control cells using only Lipofectamine 2000 were counted and plated at 2 × 10^4 ^cells in at least 6 wells of a 96-well plate. The same volume of cells used in this control at 24-hours, was used in the following treatments at that time point and the remaining 48 and 72-hour time points. After the cells were replated, an equal amount of cell viability substrate was added to each well, according to protocol. After the substrate is added, the plate was placed on a rocker for 2 mins then incubated for 10 mins. Finally, the plate was read 3 times per treatment on a Victor^3^V 1420 Multilabel counter and analyzed using the Wallac 1420 software.

## Authors' contributions

MS carried out all the biochemical analyses and cell-based assays with assistance from SY. JR performed the CD studies to determine the structure of HDG 20 and PC12 cell viability assays. HPO helped in establishing both testing systems and in conjunction with EK conceived the study, data interpretation and drafted the manuscript. All authors read and approved the final manuscript.
